# Human Botulism in France, 1875–2016

**DOI:** 10.3390/toxins12050338

**Published:** 2020-05-21

**Authors:** Christine Rasetti-Escargueil, Emmanuel Lemichez, Michel R. Popoff

**Affiliations:** Unité des Toxines Bactériennes, UMR CNRS 2001, Institut Pasteur, 75015 Paris, France; christine.rasetti-escargueil@pasteur.fr (C.R.-E.); emmanuel.lemichez@pasteur.fr (E.L.)

**Keywords:** botulism, food-borne botulism, infant botulism, *Clostridium botulinum*, botulinum neurotoxins, food poisoning

## Abstract

Botulism is a rare but severe disease which is characterized by paralysis and inhibition of secretions. Only a few cases had been reported at the end of the 19th century in France. The disease was frequent during the second world war, and then the incidence decreased progressively. However, human botulism is still present in France with 10–25 cases every year. Food-borne botulism was the main form of botulism in France, whereas infant botulism (17 cases between 2004 and 2016) was rare, and wound and inhalational botulism were exceptional. Type B was the prevalent botulism type and was mainly due to consumption of home-made or small-scale preparations of cured ham and to a lesser extent other pork meat products. In the recent period (2000–2016), a wider diversity of botulism types from various food origin including industrial foods was reported. Severe cases of type A and F botulism as well as type E botulism were more frequent. Albeit rare, the severity of botulism justifies its continued surveillance and recommendations to food industry and consumers regarding food hygiene and preservation practices.

## 1. Introduction

Botulism is a serious disease of humans and vertebrate animals that is characterized by flaccid paralysis and inhibition of secretions. The most severe cases lead to respiratory distress and death. Botulism is caused by potent neurotoxins, called botulinum neurotoxins (BoNTs), which are produced by toxigenic *Clostridium botulinum* and more rarely by atypical strains from other clostridia (*Clostridium baratii, Clostridium butyricum*) and non-*Clostridium* species. BoNTs show a high immunological and genetic diversity. Indeed, BoNTs are divided into several toxinotypes (A, B, C, D, E, F, G, H, or F/A) based on their neutralization by specific corresponding antisera. In addition, each toxinotype is subdivided into subtypes based on amino acid variation (>2.6% in most cases). Up to now 41 subtypes have been identified [1]. More recently, a novel BoNT type called BoNT/X was characterized in a *C. botulinum* strain that also produces BoNT/B2 [2]. *BoNT genes* (*bont*) related sequences have been identified in a few non-clostridial strains such as *bont*/Wo or *bont*/I from *Weisenella oryzae*, a bacterium of fermented rice, *bont*/J (e*bont*/F or *bont*/En) from an *Enterococcus faecalis* strain in a cow, and Cp1 from *Chryseobacterium piperi* from sediment [3,4,5,6,7,8]. The activity of the non-clostridial BoNTs has not been characterized. Humans and animal species show variable sensitivity to the distinct toxinotypes. Human botulism is mainly caused by BoNT/A, B, E, and to a lower extent F, whereas BoNT/C and BoNT/D are prevalent in animal botulism and extremely rare in humans [9].

Naturally acquired botulism occurs in different clinical forms. Foodborne botulism results from the ingestion of preformed BoNT in food. Foods contaminated by *C. botulinum* spores that have not been or have been minimally processed and stored in conditions allowing *C. botulinum* growth, are at risk of containing BoNTs. Botulism by intestinal colonization is due to ingestion of *C. botulinum* spores or bacteria that develop and produce BoNT in situ in the intestine. This form is more frequent in infants up to 1 year (infant botulism) and occasionally in adults. Wound botulism is caused by *C. botulinum* growth and BoNT production in a wound. Drug users are at risk of wound botulism by injection with contaminated materials or drugs. Inhalational botulism, which results from aerosolization of BoNT, is very rare. Only, a few laboratory cases have been reported. Iatrogenic botulism is related to toxin overdoses for therapeutic or cosmetic purpose [10,11,12,13]. No iatrogenic botulism cases have been reported in France during the considered period.

Botulism in France was first investigated by certain hospital laboratories and mainly by the Anaerobe Laboratory (Institut Pasteur, Paris) under the direction of A. R. Prévot. The laboratory of anaerobic bacteria of Institut Pasteur was recognized as the National Reference Center of Botulism in 1978. Since 1986, human botulism is subjected to mandatory declaration in France. Since 1992, the survey of botulism based on clinical reporting and bacteriological investigations was performed by the RNSP (Réseau National de Santé Publique), which was converted into InVS (Institut national de Veille Sanitaire) in 1998, and then in SPF (Santé Publique France) in 2016.

Up to 1971, botulism cases were defined on clinical symptoms and investigation of suspected foods including detection of BoNT by mouse bioassay (MBA) and eventually isolation and characterization of *C. botulinum.* From 1971, BoNT investigation in the serum of patients by MBA was used for the diagnosis of botulism. Since 1998, the survey of botulism is coordinated by InVS/SPF and the National Reference Center based on the clinical declarations and investigations of biological (serum, feces) and food samples including BoNT identification by MBA as well as detection of toxigenic *C. botulinum* by molecular biology method in feces/food and isolation/characterization of *C. botulinum* strains.

This review is from published scientific papers from international and national literature.

## 2. Foodborne Botulism

The first detailed clinical description of botulism was performed by Kerner in 1817–1822 in the southwest German region of Wurtenberg. The disease was associated to the consumption of pork blood sausage and was initially termed sausage poisoning. The name botulism was coined later in the second half of the 19th century from the Latin word “botulus” meaning sausage [14,15]. Botulism was not reported or misdiagnosed during this period in Alsace which is close to the Wurtenberg region and where the consumption of pork sausages was common. The bacterial origin of botulism was determined by Emile Pierre van Ermengem, professor at the university of Ghent, who isolated the first toxigenic *C. botulinum* strain from the ham and one of the victims of a severe outbreak of botulism which occurred in a small Belgian village (Ellezelles) [16].

### 2.1. Period 1875–1944

The first most likely outbreak of human botulism in France was reported in 1875. Eleven prisoners who ate canned beef developed clinical signs of botulism, four of them died. The meat can was opened, stored at room temperature, and distributed to the prisoners 5 days later [17]. Botulism was rare in France before the second world war, probably because the consumption of canned foods was not traditional, although canned food products were developed by the French inventor Nicolas Appert in 1795–1806 [18]. From 1875 to 1936, 24 cases of botulism were recorded, and eight from 1936 to 1940 [19,20]. For example, one case was reported in a 42-year old man who ate home-made hare pâté [21], and an outbreak occurred in four people after consumption of canned smoked trout [22]. In 1935, two cases of botulism occurred after consumption of canned spinach from Morocco [23].

**Figure 1:**
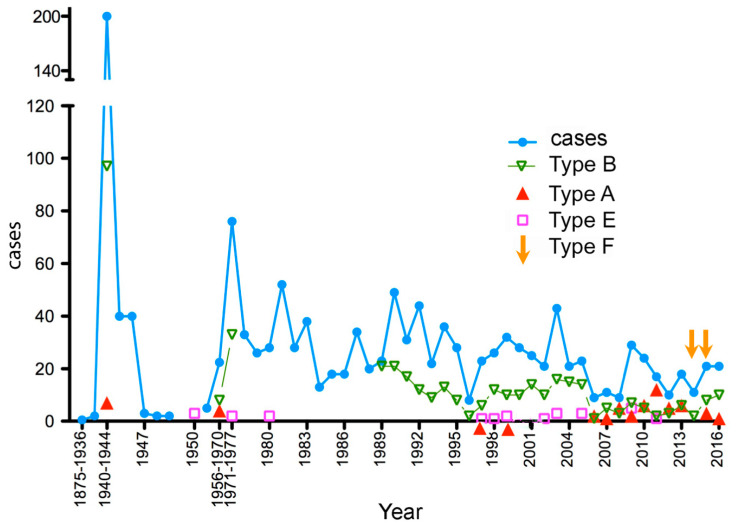
Incidence of human botulism in France, 1875–2016. The numbers indicated in the period ranges 1875–1936, 1940–1944, 1956–1970, and 1971–1977 are the annual mean values. Total cases (blue), type B botulism (green), type A botulism (red), type E botulism (purple), type F botulism (orange arrows) according to [17,19,24,25,26,27,28,29,30,31,32,33,34,35] and modified from [11].

During the second world war (1940–1944), the incidence of botulism was very high (Figure 1). More than 500 outbreaks and 1000 cases were reported in France. Food deprivation and poor hygiene in the preparation of home-made preserved foods were the main factors responsible for the high incidence. Botulism type B was the most prevalent and most of the incriminated foods (93%) were from pork meat preparations, notably cured ham (90%). Only three outbreaks of botulism type A were identified in 1943. The mortality rate was low about 2% (15 deceased) [20,24].

### 2.2. Period 1945–1970

The incidence of human botulism strongly decreased after the second world war; 40 outbreaks in 1945, 40 in 1946, three in 1947, and two in 1948 [35]. In the period 1950–1954, five outbreaks including 26 cases were recorded [19]. The first outbreak of botulism type E in France was identified in 1951. Three people of the same family developed a botulism type E after the consumption of canned sardines [36]. Another case of botulism type E was reported in a man who ingested canned tuna in a restaurant in 1952 [37].

From 1956 to 1970, 73 outbreaks (149 cases) of human botulism were investigated. Except for two outbreaks (five cases of which two were fatal) which were type A botulism subsequently to ingestion of home-made beans, 70 outbreaks were type B botulism due to home-made preparations of cured ham, and in one outbreak (one case) following consumption of asparagus the botulism type was not determined [38,39]. Additional cases of botulism have been reported based on clinical symptoms. Thus, for this period the total number of outbreaks was 134 (5.5/year) including 337 (22.4/year) cases from which 17 deaths were reported [28,38].

### 2.3. Period 1971–1986

Since 1971, the diagnosis of botulism which was based on investigation of suspected foods, was improved by detection of BoNT in the serum of patients [40]. Thereby the incidence of human botulism was increased (70.8 annual cases between 1971 and 1978) due to the improved diagnosis of botulism but possibly also to the introduction of novel foods at risk of botulism such as industrial foods. During the period 1971–1986, 394 outbreaks and 821 cases of which at least 16 deaths were reported [33,41].

Botulism type B was the most prevalent type based on investigations of food and/or patient’s sera (97%). The food origin was identified in 41% of the outbreaks and was mainly home-made preparations of pork meat (about 73%), notably cured ham, and more rarely home-made preserved vegetables. In 33 outbreaks (167 cases) from 1971 to 1979, the origin of botulism was from commercial foods including industrial cans or preparations from small scale producers, and in four outbreaks (five cases) botulism resulted from meals at restaurant. They consisted of type B botulism except one type A outbreak (one case) with pork meat and one type E outbreak (two cases) with an industrial can of crab from Russia. Most of the other outbreaks (21 outbreaks, 64 cases) resulted from pork meat preparations (ham, pie). In two outbreaks in 1972 and 1984, industrial cans of asparagus from Spain caused five type B botulism cases (three deaths) [33,42]. A defect in the industrial sterilization process in 1984 was involved in *C. botulinum* growth in the cans containing unpeeled asparagus and likely contaminated by soil. It is noteworthy that the incidence of contaminated industrial cans was low. BoNT/B and *C. botulinum* B were detected in only two cans and not in 82 other investigated cans from the same company [42]. Industrial cheese was involved in two large outbreaks (32 and 37 cases) in 1973 and 1978 and in one additional outbreak in Switzerland. The cheeses were contaminated during the maturation process of 2–3 months at 12.5 °C on straw contaminated by *C. botulinum* B [33,43]. Other incriminated foods were seafood (three outbreaks, six cases), canned pears (one outbreak, one case), paella (one outbreak, one case), and undetermined in four outbreaks (13 cases) [33]. A home-made preparation of salted herrings was responsible of one outbreak (two cases) of botulism type E in 1980 [44]. A second case of botulism type E occurred in the same year due to a fresh water fish preparation [45].

One atypical botulism type was recorded in 1972. Four persons of the same family developed clinical symptoms of botulism, one person died. BoNT type C was identified in the serum of two patients. The consumption of a smoked chicken was suspected to cause this outbreak but has not been confirmed [46]. Botulism type C is common in birds but it is exceptional in humans [9]. In 1981, a fatal case of botulism occurred in a 21-year old woman who ate a home-made canned preparation of rabbit with pork fat. The incriminated food contained both BoNT/A and BoNT/B, and a bivalent *C. botulinum* strain producing 10-fold more BoNT/A than BoNT/B was isolated [47].

### 2.4. Period 1987–2016

The survey of human botulism became more efficient in France since the mandatory declaration of clinical and laboratory confirmed cases of botulism (1986) and national recordings by the governmental organizations (RNSP from 1992, InVS in 1998, and then SPF since 2016) (see above). The incidence of human botulism progressively decreased in the period 1987–2016 compared to the previous period (Figure 1). The botulism incidence was estimated to 0.018–0.033 cases per 100,000 population during the period 2013–2016 [29]. However, every year 5–28 outbreaks (annual incidence 13.4 outbreaks/year) of food-borne botulism (9–49 cases, annual incidence 24.3 cases/year) were observed. The incidence in the last 7 years (2010–2016) (9.4 outbreaks/year; 17.4 cases/year) was about two-fold lower than that in the first 7 years of this period (1987–1993) (18.1 outbreaks/year; 31.8 cases/year) confirming the overtime decline of human botulism in France.

The clinical signs and symptoms of human botulism recorded in the period 2007–2016 are shown in Table 1. The main symptoms include oculomotor disorders (diplopia, blurred vision, mydriasis) dry mouth, dysphagia, and in the more severe cases paralysis of limbs and diaphragm. The gastrointestinal disorders (vomiting, diarrhea), which only occurred in the first days of the disease and preceded the neurological symptoms, are not specific of botulism and rather reflect other food contaminants [13]. In contrast constipation, which is often observed throughout the course of the disease is related to BoNT effect on the intestinal motility. Similar clinical symptoms were reported about 108 cases between 1966 and 1990 in the Poitou Charentes region [48] as well as in 26 observations in northern France [49]. It is noteworthy that some mild form of botulism type B resulted only of oculomotor disorders [50,51].

Botulism type B (85% of the identified botulism outbreaks) (Table 2) was the most prevalent type during the period 1987–2016. Botulism type A (9.4% of the identified outbreaks) was responsible for the most severe cases of botulism which most often results in hospitalization of the patients in intensive care units. Botulism type A was more frequent (28.4%) in the recent period 2006–2016 than in the previous periods. Two outbreaks of the rare *C. baratii* type F (F7) botulism were observed recently in 2014 and 2015 (Table 2). Type E botulism was rare (4.0% of the identified outbreaks).

The food origin of botulism was identified in less than half of the outbreaks (185, 46.0% of the outbreaks). It could not be ruled out that other forms of botulism were involved. Wound botulism was excluded in the absence of wound. Food-borne botulism or possibly botulism by intestinal colonization, which also involves an initial oral contamination most probably by food, were the most likely forms of human botulism. The identification of contaminated food was often problematic. Indeed, the incubation period and the delay of suspicion/diagnosis of clinical botulism by physicians who often have no experience in this disease, induce a late investigation of the contaminated foods. Very often there was no leftover of the food responsible for botulism; the contaminated meals being totally consumed or the leftovers being discarded. Traditional hams which consist of large pieces of meat were the most often available food for investigation. Indeed, ham from home-made preparation or from small scale producers was involved in 73.5% of the botulism outbreaks with identified food source. Ham and other pork/boar meat preparations were responsible for most of the identified botulism outbreaks (83.7%) (Table 2). These outbreaks were type B botulism, more specifically B4, except one type A outbreak from home-made pie, and one type E outbreak from ham (Table 2). In another outbreak, the ham contained both BoNT/B and BoNT/E [62]. The other sources of botulism were home-made canned vegetables or fruits (beans, asparagus, eggplant, spinach, pumpkin, chestnut) in nine outbreaks, home-made meat or fish preparations in four outbreaks, and industrial foods (fish soup, chicken/beef sausage, chicken/enchiladas, ground meat, olives/dried tomatoes, fresh pasta carbonara) in 14 outbreaks (Table 2). Most of the outbreaks with non-pork meat were type A botulism. Home-made canned vegetables (beans, asparagus, spinach) and beef/chicken sausages were responsible for three and two type B botulism outbreaks, respectively. Two outbreaks of type E botulism were due to home-made marinated fish and commercial preserved fish/sea products were involved in at least four outbreaks. A vacuum-packed hot-smoked whitefish processed in Canada, purchased by a family in Finland, and later consumed in France caused type E botulism in three persons [61]. An industrial ground meat prepared in restaurant was identified as the source of one outbreak of *C. baratii* type F botulism [68]. The origin of the other *C. baratii* type F outbreak was not identified.

## 3. Infant Botulism and Botulism by Intestinal Colonization

The first case of infant botulism was identified in France in 2004. During the period 2004–2016, 15 infant botulism cases and two botulism cases by intestinal colonization in 12- and 18-months old infants were reported (Table 3). Nine were type A botulism and eight type B. The origin of these botulism cases was not identified. All the food samples possibly involved in these cases that have been investigated were negative. Six babies received honey. However, no *C. botulinum* spores were detected in any of the six honey samples. Environmental contamination was suspected in two cases. A 2-month old girl living close to a thermal power station that intermittently releases sprays of vapor and smoke/dust, developed several relapses of botulism. *C. botulinum* A2 resistant to β-lactams was isolated on stools up to 110 days after onset [70]. In the second outbreak, a 5-month old male infant exclusively breastfed was living close to a construction site. He presented clinical symptoms of botulism and BoNT/B was detected in his stools. *C. botulinum* B was isolated from stool as well as from three soil samples of the active construction site [71]. Exposure to dust containing *C. botulinum* spores was the only risk factor possibly involved in these two infant botulism cases.

In addition, a 10-year boy with history of Meckel’s diverticulum and chronic constipation, presented dysarthria, dry mouth, hypotony, respiratory failure, and a cardiac arrest in 2011. Stool analyses were positive for *C. butyricum* E5 by PCR and DNA sequencing up to 2 months after discharge. Botulism by intestinal colonization with neurotoxigenic *C. butyricum* from undetermined origin was strongly suspected in this boy [31]. Botulism by intestinal colonization with *C. butyricum* type E was also found in two young boys having a Meckel’s diverticulum in Italy [76].

## 4. Wound Botulism and Inhalational Botulism

One case of wound botulism has been reported in a man with open leg fracture in 2008. The wound was suppurative and the patient developed signs of paralysis. BoNT/B was detected in the patient’s serum [30].

Two young men who inhaled cocaine, presented mild form of botulism. BoNT/B was identified in the serum of one of the two patients [77].

## 5. Discussion

Human botulism form and incidence are variable according to the countries and depend notably on the dietary habits. In France, food-borne botulism is the most prevalent form of botulism. In Europe, Italy has also a high incidence of human food-borne botulism [78]. In contrast to Italy where food-borne botulism is mainly caused by canned vegetables, the disease in France is mainly due to pork meat products, more specifically to cured ham, and the most prevalent form of botulism is type B. Pigs are often healthy carriers of *C. botulinum* B in their digestive tract [79,80,81,82]. Pork meat can be contaminated by intestinal *C. botulinum* spores, and preparations of uncooked and salted ham followed by storage at room temperature for several weeks or months facilitate *C. botulinum* growth and toxin production especially if the salt concentration is insufficient (lower than 5%) [83]. It is noteworthy that the *C. botulinum* strains isolated from ham were type B4 from group II (non-proteolytic strains), whereas the strains from canned vegetables were type B2 from *C. botulinum* group I (proteolytic strains). The incidence of human botulism was high at the beginning of the 20th century and then declined progressively (Figure 1). Improved sanitary conditions of ham preparation and storage likely contributed to the prevention of botulism outbreaks. Moreover, home-made preparations of ham and other pork products are less and less in use by the families in favor of industrial foods prepared in more strict safety conditions. Albeit botulism outbreaks can occur in all parts of France, type B botulism from ham and other pork meat preparations were mainly observed in the areas in the center of France where these foods are traditional [29,31].

BoNT/B concentrations in hams responsible for human botulism are variable from 4 to 60,000 mouse lethal doses (MLD)/g (Table 4). The toxin content can also differ in different parts of the same ham. For example, four samples of a same ham yielded <4, 4, 8, and 1000 MLD/g [29]. If we consider the common lowest BoNT/B concentration of 40 MLD/g (Table 2) and usual average portion of 100 g consumed by the patients, the minimal toxic dose by oral route is about 4000 MLD for an adult. Taking account of a 100-fold activation by trypsin of BoNT/B4 from *C. botulinum* group II as reported in ham sample tested by mouse bioassay before and after trypsinization [62] and of the value of 8.3 pg for 1 MLD50 of purified BoNT/B [84], the BoNT/B minimal toxic dose is estimated to 400,000 MLD or 3.2 μg by oral route to induce botulism symptoms in an adult. Minimal doses of 30–100 ng of BoNT/B have been reported to induce food-borne botulism in humans [85]. These values match our estimations without considering trypsin activation.

The origin of contamination by *C. botulinum* E of two hams responsible for two outbreaks was undetermined. A possible explanation could be the use of marine salt possibly contaminated with *C. botulinum* E spores for the preparation of the hams [31].

Food-borne botulism type A was rare in France before 1997. It was more prevalent in the recent period 1997–2013 (Figure 1). The main origin was home-made canned vegetables (asparagus, beans, eggplant, pumpkin jam) and commercial foods (fish soup, enchiladas, tapenades with olives/dried tomatoes, fresh pasta carbonara). Two outbreaks were related to home-made meat preparations (pheasant, pork meat pie) (Table 2). The commercial foods responsible for food-borne botulism were consumed after the indicated expiry date and were stored at room temperature instead of cold conditions.

Type E botulism was not usual in France, and was mainly due to imported canned or preserved fish and sea food products. Very rare atypical botulism types were observed such as type C botulism in two patients [46], type AB [47], and *C. baratii* type F in two outbreaks [67,68]. Interestingly, an atypical non-neurotoxigenic *Clostridium* strain related to group III *C. botulinum* D/C was isolated from the blood of a 83-year old patient who died from a sepsis syndrome without the characteristic symptom of flaccid paralysis in 2010 [86].

Botulism outbreaks in France commonly concerned a low number of patients by outbreak. Independent unique cases are considered as distinct outbreaks including one case each. The size of the botulism outbreaks ranged from 1 to 6 as observed in the period 1998–2016 and most of the outbreaks (72%) consisted of only one patient (Table 5). The two largest outbreaks involved 32 and 37 cases and were due to industrial cheese [33,43].

Infant botulism in France was rare (17 cases from 2004 to 2016) compared to the US (110–150 cases/year) [87] and Italy (36 cases between 1986 and 2015) [78]. The origin of infant botulism was undetermined except in two cases where environmental contamination was strongly suspected. It is not precluded that a higher number of undiagnosed cases could occur. Indeed, infant botulism is a rare disease and sometimes the pediatricians did not suspect this disease even in the presence of characteristic signs of paralysis and constipation. Moreover, some of them ruled out a diagnosis of infant botulism when the babies did not have honey, since they considered that honey was the unique source of contamination.

The other forms of botulism were exceptional in France. One wound botulism case and two botulism cases by inhalation in drug users were reported [30,77].

Food-borne botulism is the most severe food-borne poisoning and represents a public health concern in France. Albeit the incidence of food-borne botulism declined since the second world war which induced a high number of cases due to poor safety conditions of food preparations, botulism cases were still observed in the recent period. Home-made and small-scale preparations of cured ham were the main source of food-borne type B botulism in France. However, in the recent periods, a wider diversity of botulism types and food origin, including industrial products, were reported. Severe cases of botulism type A and F, as well as botulism type E were more frequent since 2000. It is noteworthy that the overall mortality of human botulism in France is low and was decreasing over time likely due to the predominance of botulism type B that is less severe than botulism types A and E as well as to better medical management including mechanical ventilation in the recent period (Table 6). Albeit rare, the severity of botulism justifies its continued surveillance and recommendations to industries and consumers regarding hygiene and food preservation practices.

## Figures and Tables

**Table 1:** Clinical signs and symptoms reported by patients with food-borne botulism, France 2007–2016 [29,30,31].

Clinical Sign/Symptom	Number of Cases/Total Cases	% of Cases
Gastrointestinal disorders		
Dry mouth	70/133	52.6
Vomiting	64/134	47.8
Constipation	62/135	45.9
Diarrhea	32/139	23.0
Nausea	22/133	16.5
Abdominal pain	20/130	15.4
Oculomotor disorders		
Diplopia	83/146	56.8
Blurred vision	63/139	45.3
Mydriasis	55/146	37.7
Ptosis	30/137	21.9
Paralysis		
Dysphagia	91/134	67.9
Food choking	54/129	41.9
Diaphragmatic paralysis	36/130	27.7
Limb paralysis	33/126	26.2
Dysarthria	20/122	16.4

**Table 2:** Human botulism in France 1987–2016.

					Outbreaks Where Food Was Identified			
Year	Outbreaks	Cases	Deaths	Type (Outbreaks)	Home-Made Ham or Ham from Small Scale Producers	Other Pork Meat Preparations Home-Made or from Small Scale Producers	Other Food (Outbreaks)	References
1987	20	34	1	B (29)	18	5		[52]
1988	15	20	0	E (1)	1 (ham from Portugal)			
1989	18	23	0	B (43) A (1)	27	8		[53]
1990	28	49	0				home-made canned asparagus (1)	
1991	19	31	0	B (29)	13 confirmed 3 suspected	3 suspected	home-made beans (1)	[28,54]
1992	15	44	0					
1993	12	22	1	B (9)	28 confirmed or suspected	15 (suspected not confirmed)		[28]
1994	14	36	0	B (13)				
1995	10	28	1	B (8)				
1996	5	8	0	B (2)				
1997	14	23	1	B (6)	4			[28,55,56,57]
				A (2)			home-made beans (2)	
				E (2)			scallops (2)	
1998	17	26	0	B (11)	2			[26,28,56,58]
				E (1)			home-made marinated fish (1)	
1999	21	32	2	B (13)	2		industrial chicken sausage (1)	
				A (2)			industrial fish soup (1)	
				AB (1)			industrial blood sausage? (1)	
				E (2)			marinated fish (1), restaurant? (1)	
2000	15	28	0	B (10)	1			
2001	18	29	0	B (14)	4			[27]
2002	17	33	0	B (11)	4			
				E (1)			chestnut jam (1)	
2003	22	43	0	B (16)	4	home-made pork meat preparation (1)	industrial beef sausage (3)	[25,59]
				E (3)			canned sardines? (1)	
2004	13	21	0	B (12)	2			
2005	16	23	0	B (14)	5	home-made pie (1)	boar meat preparation (1)	
2006	5	9	0	B (1)				
				A (1)		home-made pie (1)		
2007	6	11	0	B (5)	2			[30,60,61,62]
				A (1)				
2008	6	9	0	B (3)				
				A (2)			industrial enchiladas with chicken meat (1) subtype A7 home-made pumpkin jam (1) subtype A1(F)	
2009	12	27	0	B (7)	2 ^1^		boar meat preparation (1)	
				A (2)				
				E (1)			vacuum-packed hot-smoked whitefish (1)	
2010	7	24	1	B (5)	4		home-made canned asparagus (1) subtype B2	[31,63,64]
				A (2)			home-made beans (1) subtype A2	
2011	9	17	0	B (2)			home-made canned spinach (1) subtype B2	
				A (5)			commercial tapenades with olives and dried tomatoes (2) subtype A1(B) industrial fresh pasta carbonara (1) subtype A5	
				E (1)				
2012	8	10	0	B (3)	2	industrial pie (1)		
				A (4)			home-made canned eggplant (1) subtype A1(B)	
2013	11	18	0	B (6)	2, subtype B4	chorizo (Portugal) (1)		[29,65,66,67,68,69]
				A (4)				
2014	4	11	0	B (2)	1			
				F (1)				
2015	14	21	1	B (8)	2, subtype B4	pie (Poland) (1) subtype B4 chorizo (Portugal) (1)		
				A (1)			home-made pheasant pâté (1) subtype A1	
				F (1)			industrial ground meat, Bolognese sauce (1) subtype F7	
2016	13	21	1	B (10)	4, subtype B4			
				A (1)				
Total	402	731	9	B (254)	B (136)	B (19 confirmed, 18 suspected)	home-made food (5) industrial food (4)	
				A (28)		A (1)	home-made food (6) industrial food (5)	
				AB (2)			industrial food (1)	
				E (12)	E (2)		home-made food (2) industrial food (4)	
				F (2)			industrial food (1)	

^1^ A ham involved in an outbreak (2 cases) contained both BoNT/B and BoNT/E [62].

**Table 3:** Infant botulism and botulism by intestinal colonization. Infant botulism cases in France from 2004 to 2016 according to [29,31,70,71,72,73,74,75].

Year	Sex	Age	Toxin in Serum	Toxin in Stool	Toxin in Stool Titer MLD/g	*C. botulinum* in Stool
2004	F	17 d	B	nd	nd	A1
2005	F	6 m	B	nd	nd	B5
2006	F	2 m	A	A	80	A2
2007	F	5 m	A	A	nd	A2
2008	F	4 m	B	B	20	culture and PCR detection negative
2009	F	2.5 m	A	A	1000	A1(B)
2009	F	18 m	neg	neg		B2
2009	F	6 m	neg	A	20	A2
2011	F	2.5 m	A	A	100,000	A2
2011	F	12 m	B	B	600	B2
2012	F	4 m	neg	A	7000	A1(B)
2013	F	1 m	neg	B	12,000	Bf2
2013	F	3 m	neg	A	4800–300,000 ^1^	A2 β-lactam resistant
2013	M	6 m	neg	B	340	Bf
2013	M	6 m	A	A	10,240	A2
2015	M	2 m	neg	B	6000	B5
2016	F	6 m	neg	B	2000	B2

^1^ several titrations during the course of the disease. nd, not done. neg, negative.

**Table 4:** BoNT/B concentration in ham (mouse lethal dose (MLD)/g) responsible for human botulism (2003–2016). *Clostridium botulinum* B4 was identified in these samples [25,29,30,31].

BoNT/B (MLD/g)	Number of Ham Samples (Total 26)
60,000	1
40,000	1
20,000	4
12,000	1
10,000	1
8000	1
5000	1
4000	1
3000	1
2000	1
1000	1
400	1
300	1
200	2
140	1
40	4
20	1
10	1
4	1

**Table 5:** Size of human botulism outbreaks in France (1998–2016, 235 outbreaks) [27,28,29,30,31].

Number of Cases/Outbreak	Number of Outbreaks
6	5
5	2
4	4
3	21
2	33
1	170

**Table 6:** Botulism mortality in the period 1956–2016 [26,27,28,29,30,31,33,41,57].

Years	Number of Cases	Deaths	% Death
1956–1970	337	17	5.0
1971–1980	621	16	2.6
1981–1990	293	12	4.1
1991–2000	278	5	1.8
2001–2016	317	3	0.9
